# Supplementary material for: The tumour microenvironment creates a niche for the self-renewal of tumour-promoting macrophages in colon adenoma
Source: Nat Commun. 2018 Feb 8;9:582. doi: 10.1038/s41467-018-02834-8 (PMC5805689; doi:10.1038/s41467-018-02834-8)
Supplement: Supplementary file 1 — Supplementary Information [file 41467_2018_2834_MOESM1_ESM.pdf]

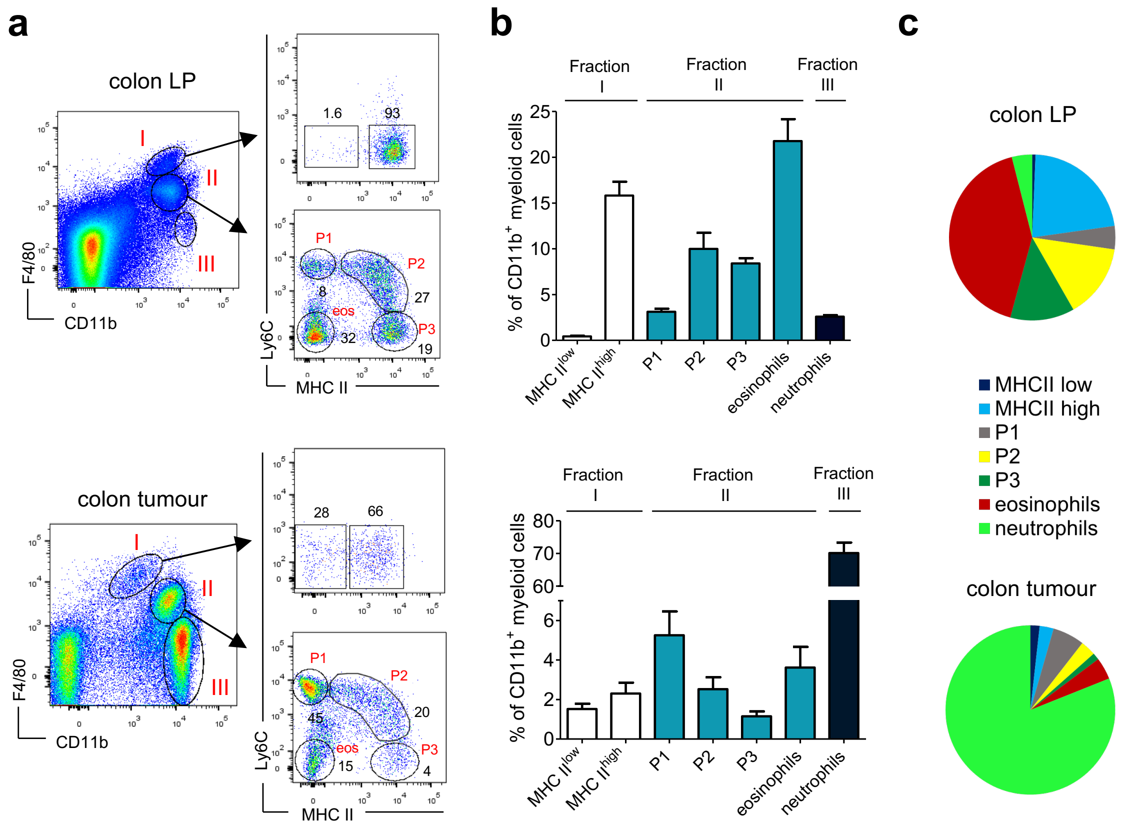

Supplementary Fig. 1: Myeloid cell heterogeneity in colon LP and spontaneous colon tumours. Tumour myeloid cell subpopulations were analysed in spontaneous colon adenomas obtained from 5 months old *Apc*$^{Min/+}$ mice and were compared to colon LP cells of aged-matched WT C57BL/6 mice (a) Flow cytometry representative dot plots of colon LP (upper panel) and tumour cell subpopulations (lower panel). Three different myeloid fractions (I-III) are defined by the differential expression of F4/80. Fraction I represents F4/80^{hi} tissue-resident macrophages, which can be further subdivided into MHCII^{high} and MHCII^{low}. Fraction II contains monocytes (P1), two monocyte-derived macrophage subpopulations (P2 and P3) and eosinophils (eos), based on differential expression of MHCII and Ly6C. Fraction III consists of neutrophils. (b) Bar charts of the distinct myeloid cell subpopulations obtained from the colon LP (n=7) (upper bar chart) and 2-3 mm colon tumours (n=8) (lower bar chart). White bars: Fraction I; light blue bars: Fraction II and black bars: Fraction III. Error bars represent the s.e.m. (c) Pie charts showing the proportions of F4/80^{hi} tissue-resident macrophages (MHCII^{high} and MHCII^{low}), monocytes (P1), monocyte-derived macrophages (P2-P3), neutrophils and eosinophils across colon lamina propria and tumours.

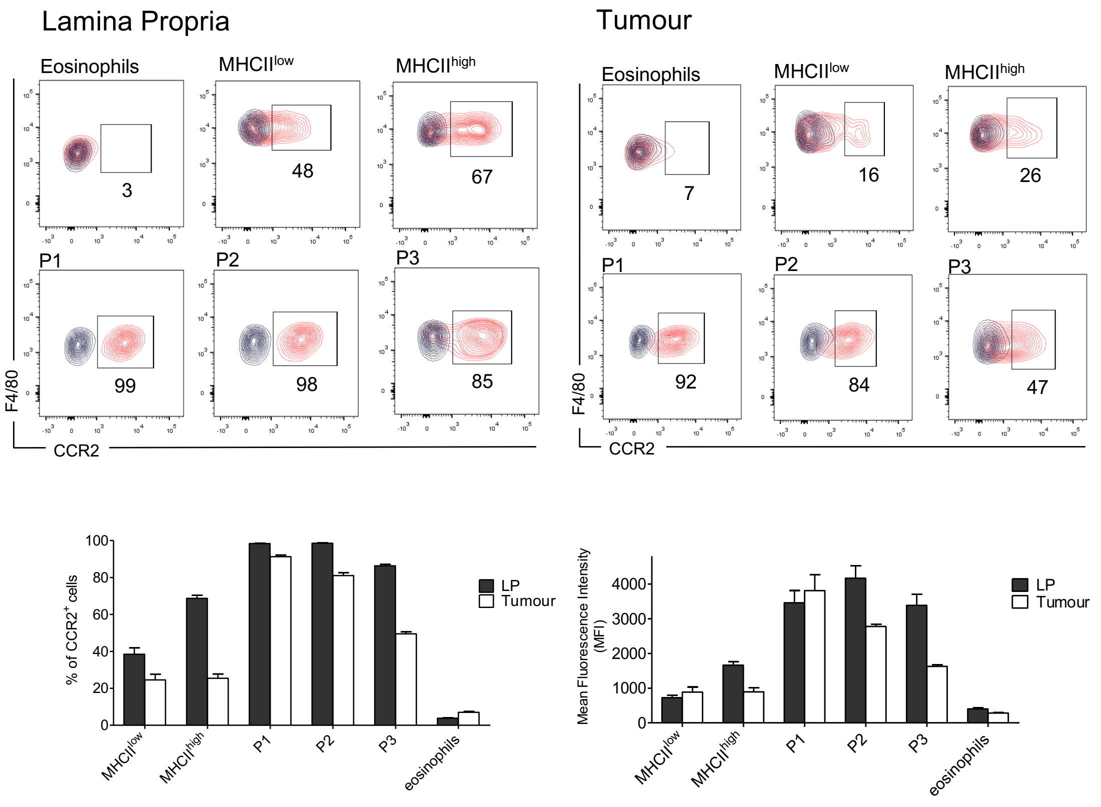

Supplementary Fig. 2: CCR2 expression pattern of distinct colon LP and intratumoural myeloid cell subpopulations. Representative flow cytometry analysis (upper panel) and bar charts representing the CCR2 expression levels (Mean Fluorescence Intensity (MFI), right panel) and % of CCR2 expressing cells (left panel). Black bars: LP and white bars: colon tumour. Total 6 LP and 6 colon tumours were analysed. Error bars represent s.e.m.

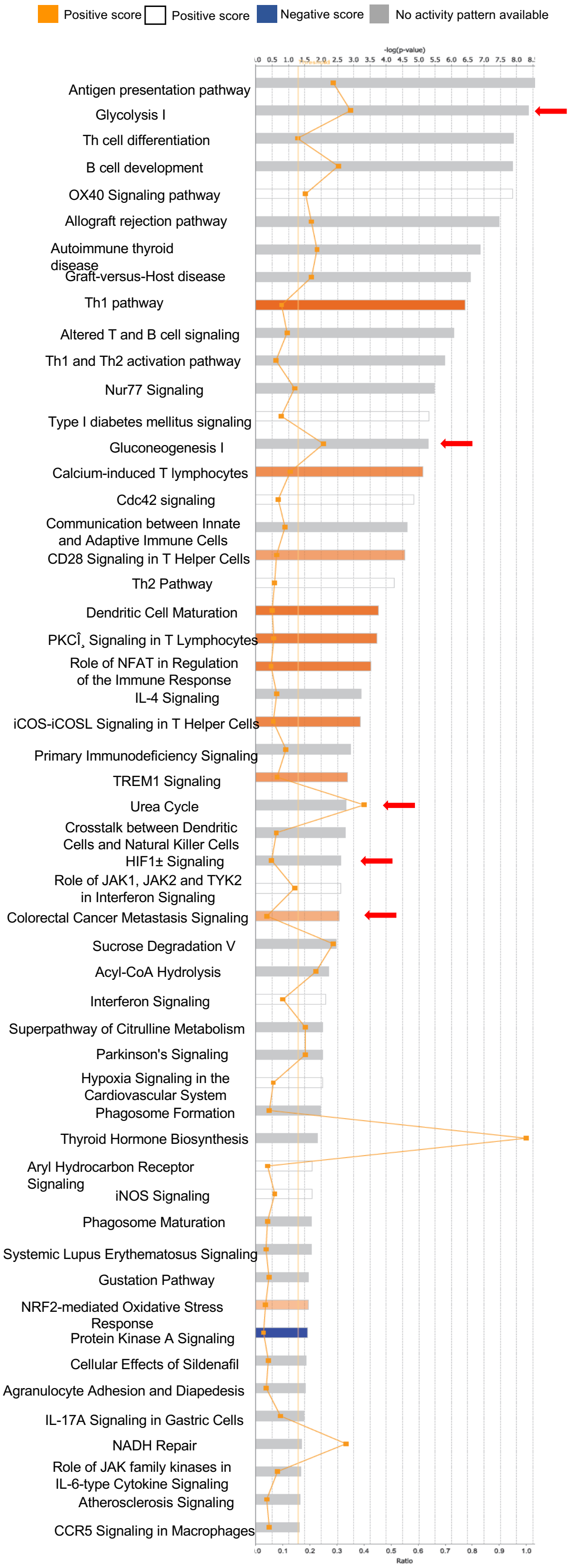

Ingenuity Canonical Pathways	-log(p-value)	Ratio	z-score	Molecules
Antigen Presentation Pathway	8.69E+00	2.86E-01	NaN	HLA-DMB,CIITA,HLA-DQA1,HLA-DOB,HLA-DQB1,CD74,HLA-DRB5,HLA-E
Glycolysis I	8.37E+00	3.50E-01	NaN	PGK1,ENO1,TPI1,PGAM1,PKM,GAPDH,ALDOA
T Helper Cell Differentiation	7.90E+00	1.54E-01	NaN	HLA-DMB,IL6R,HLA-DQA1,CD86,HLA-DOB,IFNGR1,HLA-DQB1,RORC,HLA-DRB5,IL17A
B Cell Development	7.88E+00	3.04E-01	NaN	PTPRC,HLA-DMB,HLA-DQA1,CD86,HLA-DOB,HLA-DQB1,HLA-DRB5
OX40 Signaling Pathway	7.87E+00	1.84E-01	NaN	JUN,H2-M2,CD4,HLA-DMB,HLA-DQA1,HLA-DOB,HLA-DQB1,HLA-DRB5,HLA-E
Allograft Rejection Signaling	7.45E+00	2.05E-01	NaN	H2-M2,HLA-DMB,HLA-DQA1,CD86,HLA-DOB,HLA-DQB1,HLA-DRB5,HLA-E
Autoimmune Thyroid Disease Signaling	6.89E+00	2.26E-01	NaN	HLA-DMB,HLA-DQA1,CD86,HLA-DOB,HLA-DQB1,HLA-DRB5,HLA-E
Graft-versus-Host Disease Signaling	6.59E+00	2.06E-01	NaN	HLA-DMB,HLA-DQA1,CD86,HLA-DOB,HLA-DQB1,HLA-DRB5,HLA-E
Th1 Pathway	6.41E+00	9.48E-02	3.317	JAK1,CD4,HLA-DMB,IL6R,HLA-DQA1,CD86,HLA-DOB,IFNGR1,HLA-DQB1,IFNAR1,HLA-DRB5
Altered T Cell and B Cell Signaling in Rheumatoid Arthritis	6.06E+00	1.15E-01	NaN	HLA-DMB,HLA-DQA1,TLR7,CD86,HLA-DOB,HLA-DQB1,HLA-DRB5,IL17A,Tlr12
Th1 and Th2 Activation Pathway	5.78E+00	7.41E-02	NaN	JAK1,JUN,CD4,HLA-DMB,IL6R,HLA-DQA1,CD86,HLA-DOB,IFNGR1, HLA-DQB1,IFNAR1,HLA-DRB5
Nur77 Signaling in T Lymphocytes	5.46E+00	1.43E-01	NaN	HLA-DMB,HLA-DQA1,CD86,HLA-DOB,HLA-DQB1,HLA-DRB5,EP300
Type I Diabetes Mellitus Signaling	5.30E+00	9.38E-02	NaN	JAK1,HLA-DMB,HLA-DQA1,CD86,HLA-DOB,IFNGR1,HLA-DQB1,HLA-DRB5,HLA-E
Gluconeogenesis I	5.29E+00	2.50E-01	NaN	PGK1,ENO1,PGAM1,GAPDH,ALDOA
Calcium-induced T Lymphocyte Apoptosis	5.12E+00	1.27E-01	2.449	CD4,HLA-DMB,HLA-DQA1,HLA-DOB,HLA-DQB1,HLA-DRB5,EP300
Cdc42 Signaling	4.85E+00	8.26E-02	NaN	JUN,H2-M2,HLA-DMB,HLA-DQA1,HLA-DOB,HLA-DQB1,HLA-DRB5,HLA-E,ITGA4
Communication between Innate and Adaptive Immune Cells	4.63E+00	1.08E-01	NaN	CD4,TLR7,CD86,CD83,HLA-DRB5,HLA-E,Tlr12
CD28 Signaling in T Helper Cells	4.57E+00	7.63E-02	2	PTPRC,JUN,CD4,HLA-DMB,HLA-DQA1,CD86,HLA-DOB,HLA-DQB1,HLA-DRB5
Th2 Pathway	4.24E+00	6.92E-02	NaN	JAK1,JUN,CD4,HLA-DMB,HLA-DQA1,CD86,HLA-DOB,HLA-DQB1,HLA-DRB5
Dendritic Cell Maturation	3.76E+00	6.00E-02	3	HLA-DMB,HLA-DQA1,CD86,HLA-DOB,CD83,HLA-DQB1,IFNAR1,HLA-DRB5,EP300
PKCÎ¸ Signaling in T Lymphocytes	3.71E+00	6.67E-02	2.828	JUN,CD4,HLA-DMB,HLA-DQA1,CD86,HLA-DOB,HLA-DQB1,HLA-DRB5
Role of NFAT in Regulation of the Immune Response	3.53E+00	5.59E-02	2.828	GNG11,JUN,CD4,HLA-DMB,HLA-DQA1,CD86,HLA-DOB,HLA-DQB1,HLA-DRB5
IL-4 Signaling	3.23E+00	7.69E-02	NaN	JAK1,HLA-DMB,HLA-DQA1,HLA-DOB,HLA-DQB1,HLA-DRB5
iCOS-iCOSL Signaling in T Helper Cells	3.21E+00	6.42E-02	2.646	PTPRC,CD4,HLA-DMB,HLA-DQA1,HLA-DOB,HLA-DQB1,HLA-DRB5
Primary Immunodeficiency Signaling	2.90E+00	1.11E-01	NaN	PTPRC,DCLRE1C,CD4,CIITA
TREM1 Signaling	2.80E+00	7.81E-02	2.236	CIITA,TLR7,CD86,CD83,Tlr12
Urea Cycle	2.77E+00	4.00E-01	NaN	ASS1,ARG1
Crosstalk between Dendritic Cells and Natural Killer Cells	2.74E+00	7.58E-02	NaN	TLR7,CD86,CD83,HLA-DRB5,HLA-E
HIF1Î± Signaling	2.61E+00	5.83E-02	NaN	JUN,MAPK6,LDHA,MMP12,ARNT,EP300
Role of JAK1, JAK2 and TYK2 in Interferon Signaling	2.59E+00	1.43E-01	NaN	JAK1,IFNGR1,IFNAR1
Colorectal Cancer Metastasis Signaling	2.56E+00	4.07E-02	1.667	JAK1,GNG11,JUN,IL6R,TLR7,IFNGR1,MMP12,ADCY7,Tlr12
Sucrose Degradation V (Mammalian)	2.45E+00	2.86E-01	NaN	TPI1,ALDOA
Acyl-CoA Hydrolysis	2.23E+00	2.22E-01	NaN	ACOT4,ACOT7
Interferon Signaling	2.14E+00	1.00E-01	NaN	JAK1,IFNGR1,IFNAR1
Superpathway of Citrulline Metabolism	2.05E+00	1.82E-01	NaN	ASS1,ARG1
Parkinson's Signaling	2.05E+00	1.82E-01	NaN	UCHL1,PARK7
Hypoxia Signaling in the Cardiovascular System	2.04E+00	6.45E-02	NaN	JUN,LDHA,ARNT,EP300
Phagosome Formation	1.97E+00	5.00E-02	NaN	MRC1,TLR7,MARCO,ITGA4,Tlr12
Thyroid Hormone Biosynthesis	1.88E+00	1.00E+00	NaN	CTSD
Aryl Hydrocarbon Receptor Signaling	1.72E+00	4.31E-02	NaN	CTSD,JUN,NFE2L2,ARNT,EP300
iNOS Signaling	1.72E+00	6.98E-02	NaN	JAK1,JUN,IFNGR1
Phagosome Maturation	1.70E+00	4.27E-02	NaN	CTSD,TUBA1A,PRDX1,TUBB,HLA-DRB5
Systemic Lupus Erythematosus Signaling	1.69E+00	3.73E-02	NaN	PTPRC,JUN,IL6R,TLR7,CD86,HLA-E
Gustation Pathway	1.61E+00	4.82E-02	NaN	GNG11,P2RX4,PDE4B,ADCY7
NRF2-mediated Oxidative Stress Response	1.61E+00	3.57E-02	1.342	JUN,PRDX1,DNAJB4,JUNB,NFE2L2,EP300
Protein Kinase A Signaling	1.58E+00	2.83E-02	-0.333	PTPRC,MYH10,GNG11,DUSP1,LIPE,PYGL,PDE4B,ADCY7,EP300
Cellular Effects of Sildenafil	1.55E+00	4.60E-02	NaN	MYH10,KCNN4,PDE4B,ADCY7
Agranulocyte Adhesion and Diapedesis	1.52E+00	3.82E-02	NaN	MYH10,VCAM1,Ppbp,MMP12,ITGA4
IL-17A Signaling in Gastric Cells	1.47E+00	9.09E-02	NaN	JUN,IL17A
NADH Repair	1.41E+00	3.33E-01	NaN	GAPDH
Role of JAK family kinases in IL-6-type Cytokine Signaling	1.37E+00	8.00E-02	NaN	JAK1,IL6R
Atherosclerosis Signaling	1.36E+00	4.00E-02	NaN	PLBD1,VCAM1,CCR2,ITGA4
CCR5 Signaling in Macrophages	1.33E+00	4.92E-02	NaN	GNG11, JUN,CD4

Supplementary Fig. 3: Ingenuity® pathway analysis (IPA®) of differentially expressed genes between intratumoural F4/80^{hi}MHCII^{hi} and F4/80^{hi}MHCII^{lo} macrophages. Bars on the left indicate the *P* values ($-\log_{10}$) for pathway enrichment (axis on the top). The pathways on the bar plots are all statistically significantly enriched pathways with p-value < 0.05 by right-tailed Fisher's exact test (-log10 p-value > 1.301). Red arrows highlight pathways involved in glycolysis, gluconeogenesis, urea cycle and colorectal cancer metastasis signalling. On the right, detailed information of the enriched pathways.

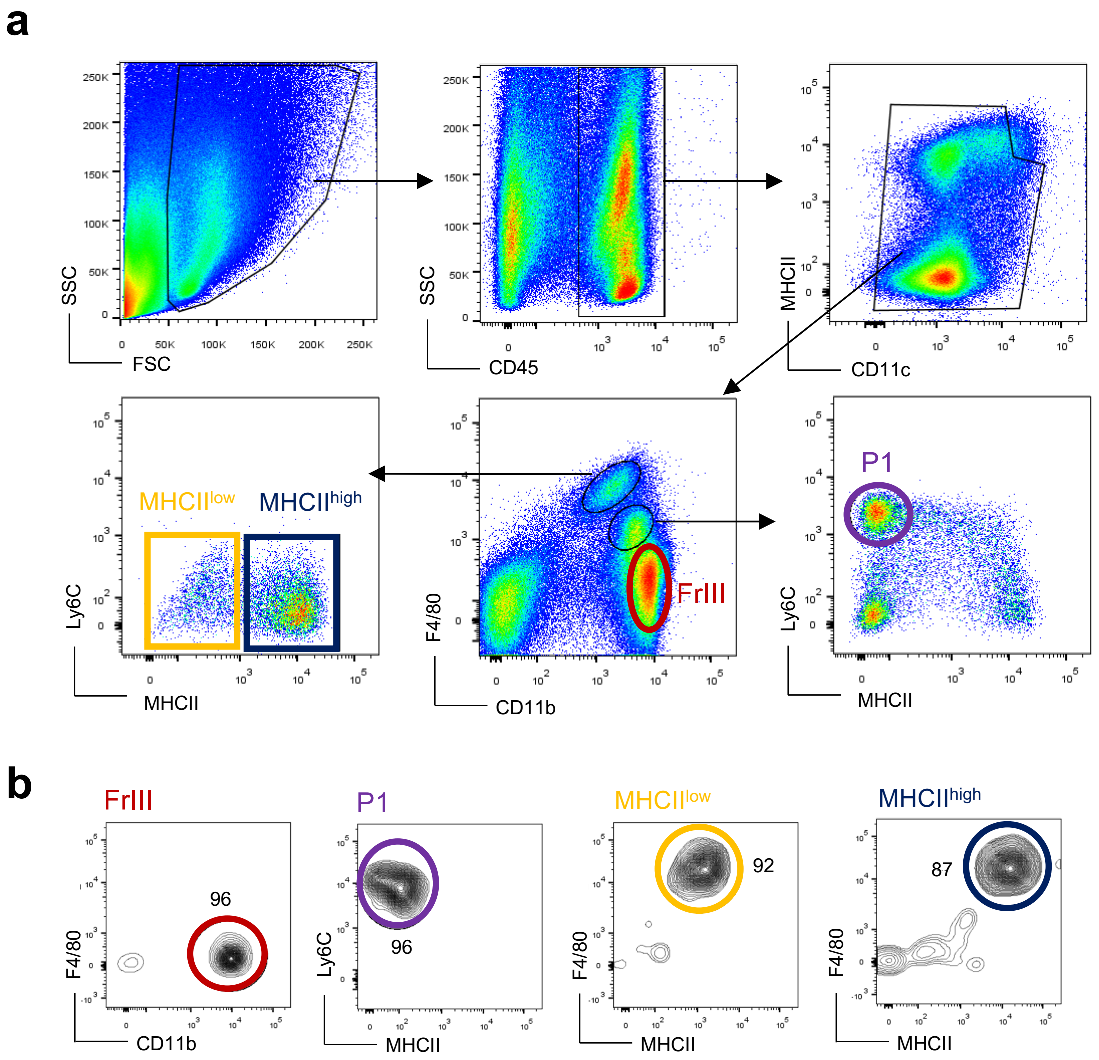

Supplementary Fig. 4: (a) **Gating strategy applied for cell sorting.** Yellow gate: F4/80^{hi}CD11b^{+}MHCII^{high}; Blue gate: F4/80^{hi}CD11b^{+}MHCII^{low}; purple gate: F4/80^{int}CD11b^{+}Ly6C^{hi}MHCII^{-} monocytes; red gate: F4/80^{-}CD11b^{+} neutrophils. (b) Purity of sorted fractions.
